# Supplementary material for: Tissue-specific regulation of PNPLA3 promotes lipid remodeling in response to dietary and environmental challenges
Source: J Hepatol. Author manuscript; Available in PMC 2026 Jul 24. (PMC13399055; doi:10.1016/j.jhep.2026.02.029)
Supplement: CTAT [file NIHMS2188109-supplement-CTAT.docx]

**Journal of Hepatology**

**CTAT methods**

Tables for a “Complete, Transparent, Accurate and Timely account” (CTAT) are now mandatory for all revised submissions. The aim is to enhance the reproducibility of methods.

- Only include the parts relevant to your study
- Refer to the CTAT in the main text as ‘Supplementary CTAT Table’
- Do not add subheadings
- Add as many rows as needed to include all information
- Only include one item per row

**If the CTAT form is not relevant to your study, please outline the reasons why:**

|  |
| --- |

- 1. **Antibodies**

| **Name** | **Citation** | **Supplier** | **Cat no.** | **Clone no.** |
| --- | --- | --- | --- | --- |
| ABHD5 |  | Novus | H00051099-M01 |  |
| AKT |  | Cell Signaling Technology | 9272 |  |
| ATGL |  | Cell Signaling Technology | 2138 |  |
| Calnexin |  | Enzo Life Sciences | ADI-SPA-860-F |  |
| G0S2 |  | Proteintech | 12091-1-AP |  |
| HA |  | Biolegend | 901513 |  |
| HSL |  | Cell Signaling Technology | 18381 |  |
| P-4EBP1 |  | Cell Signaling Technology | 9451 |  |
| P-AKT473 |  | Cell Signaling Technology | 4060 |  |
| Phospho-(Ser/Thr) PKA |  | Cell Signaling Technology | 9621 |  |
| P-HSL563 |  | Cell Signaling Technology | 4139 |  |
| PLIN1 |  | Cell Signaling Technology | 3470 |  |
| PLIN2 |  | Abcam | ab108323 |  |
| P70S6K |  | Cell Signaling Technology | 9202 |  |
| P-P70S6K |  | Cell Signaling Technology | 9205 |  |
| PPARg |  | Cell Signaling Technology | 2435 |  |
| P-ULK1 |  | Cell Signaling Technology | 5869 |  |
| PNPLA3 (19A6) | PMID: 28520213 | Lab made | N/A | 19A6 |
| RPL22 |  | Santa Cruz | sc-522583 |  |
| RPL7 |  | Novus Biologicals | NB100-2269 |  |
| RPS6 |  | Cell Signaling Technology | 2217 |  |
| Ubiquitin |  | Cell Signaling Technology | 43124 |  |
| V5 |  | Thermo Fisher Scientific | R960-25 |  |
| Peroxidase AffiniPure Goat Anti-Rabbit IgG (H+L) |  | Jackson Immunoresearch Laboratories | 111-035-144 |  |
| Peroxidase AffiniPure Donkey Anti-Mouse IgG (H+L) |  | Jackson Immunoresearch Laboratories | 715-035-150 |  |
| [Rabbit TrueBlot®: Anti-Rabbit IgG HRP](https://inventory.labarchives.com/inventory/1009147) |  | Rockland Immunochemicals, Inc. | 18-8816-33 |  |
| [Mouse TrueBlot® ULTRA: Anti-Mouse Ig HRP](https://inventory.labarchives.com/inventory/1009146) |  | Rockland Immunochemicals, Inc. | 18-8817-33 |  |

- 1. **Cell lines**

| **Name** | **Citation** | **Supplier** | **Cat no.** | **Passage no.** | **Authentication test method** |
| --- | --- | --- | --- | --- | --- |
| 3T3-L1 murine fibroblasts | PMID: 32730227 | ATCC | CL-173 | <10 |  |

- 1. **Organisms**

| **Name** | **Citation** | **Supplier** | **Strain** | **Sex** | **Age** | **Overall n number** |
| --- | --- | --- | --- | --- | --- | --- |
| Mouse: *Pnpla3^-/-^* | PMID: 29555681 | N/A | C57BL/6J | Male and Female | 12-18 weeks | 100 |
| Mouse: *Pnpla3* 148M knock-in (*Pnpla3^M/M^*) | PMID: 29555681 | N/A | C57BL/6J | Male and Female | 12-18 weeks | 60 |
| Mouse: *Pnpla3* 47A knock-in (*Pnpla3^A/A^*) | PMID: 29555681 | N/A | C57BL/6J | Male and Female | 12-18 weeks | 18 |
| *Rpl22-HA^fl/f^*^l^ Tg (*Adipoq*-Cre) | This paper | N/A | C57BL/6J | Male | 12-18 weeks | 24 |

- 1. **Sequence based reagents**

| **Name** | **Sequence** | **Supplier** |
| --- | --- | --- |
| qPCR primers: mouse HPRT | CCTCATGGACTGATTATGGACAG;  AATCCAGCAGGTCAGCAAAG | Integrated DNA Technologies IDT |
| qPCR primers: mouse Cyclophilin B | TGGAGAGCACCAAGACAGACA;  TGCCGGAGTCGACAATGAT | Integrated DNA Technologies IDT |
| qPCR primers: mouse PNPLA3 | CGAGGCGAGCGGTACGT;  TGACACCGTGATGGTGGTTT | Integrated DNA Technologies IDT |
| qPCR primers: mouse ABHD5 | AATGTGTCCCCTGCACTTACAA;  GAACATCAGCGTCCATATTCTGTT | Integrated DNA Technologies IDT |
| qPCR primers: mouse ATGL | GAGAGAACGTCATCATATCCCACTT;  CCACAGTACACCGGGATAAATGT | Integrated DNA Technologies IDT |
| qPCR primers: mouse UCP1 | ACTGCCACACCTCCAGTCATT;  CTTTGCCTCACTCAGGATTGG | Integrated DNA Technologies IDT |
| qPCR primers: mouse PLIN1 | GGTGAGCGGGACCTGTGA;  TTCTCATAGGCATTGCACACAGA | Integrated DNA Technologies IDT |
| qPCR primers: mouse U6 | GTGCTCGCTTCGGCAGC;  AAAAATATGGAACGCTTCACGAAT | Integrated DNA Technologies IDT |
| qPCR primers: mouse HSL | GGAGCACTACAAACGCAACGA;  TCGGCCACCGGTAAAGAG | Integrated DNA Technologies IDT |
| qPCR primers: mouse Co3 | CCAAGGCCACCACACTCCTA;  GGTCAGCAGCCTCCTAGATCA | Integrated DNA Technologies IDT |
| qPCR primers: mouse ND1 | GCTTTACGAGCCGTAGCCCA;  GGGTCAGGCTGGCAGAAGTAA | Integrated DNA Technologies IDT |

- 1. **Biological samples**

| **Description** | **Source** | **Identifier** |
| --- | --- | --- |
|  |  |  |

- 1. **Deposited data**

| **Name of repository** | **Identifier** | **Link** |
| --- | --- | --- |
|  |  |  |

- 1. **Software**

| **Software name** | **Manufacturer** | **Version** |
| --- | --- | --- |
| Image Studio Lite | LI-COR | v5.2 (version) |
| Prism 10 | GraphPad | 10.2.3 (version) |

- 1. **Other (e.g. drugs, proteins, vectors etc.)**

| **Reagent or Resource** | **Source** | **Identifier** |
| --- | --- | --- |
| Ad-RR5 | PMID: 20034933 | N/A |
| Ad-PNPLA3(WT) | PMID: 20034933 | N/A |
| 4X Laemmli Sample Buffer | Bio-Rad | 1610747 |
| 6X Laemmli Sample Buffer | Thermo Fisher Scientific | J61337.AC |
| TRIS Buffered Saline (TBS) | Sigma-Aldrich | T6664 |
| HEPES | Thermo Fisher Scientific | 15630080 |
| [PBS, pH 7.4](https://inventory.labarchives.com/inventory/1267777) | Gibco | 10010023 |
| [RNA Gel Buffer (10X MOPS Buffer)](https://inventory.labarchives.com/inventory/1101368) | Fisher Scientific | 50-983-261 |
| [RIPA Lysis and Extraction Buffer](https://inventory.labarchives.com/inventory/1343530) | Thermo Fisher Scientific | 89900 |
| Fetal Bovine Serum (FBS) | Millipore Sigma | F0926 |
| Bovine Serum Albumin (BSA), Cohn Fraction V | Avantor | J64944-22 |
| cOmplete Mini EDTA-free Protease Inhibitor Cocktail | Sigma-Aldrich | 11836170001 |
| Dimethyl Sulfoxide (DMSO) | Sigma-Aldrich | D2650 |
| Glycerol | Sigma-Aldrich | G9012 |
| Penicillin-Streptomycin | Corning | 30-002-Cl |
| MG132 | Peptide Institute, INC | 3178-v |
| [LY294002](https://inventory.labarchives.com/inventory/1165758) | Selleck Chemicals | S1105 |
| [AKT inhibitor VIII](https://inventory.labarchives.com/inventory/1173961) | MedChemExpress | 612847-09-3 |
| [Torin 1](https://inventory.labarchives.com/inventory/1173942) | Selleck Chemicals | S2827 |
| [Rapamycin](https://inventory.labarchives.com/inventory/1214689) | Sigma-Aldrich | 553211 |
| Forskolin | Millipore Sigma | F6886 |
| 8-Bromo-cAMP | Selleckchem | S7857 |
| [H-89 dihydrochloride hydrate](https://inventory.labarchives.com/inventory/1403500) | Millipore Sigma | B1427 |
| [Cycloheximide](https://inventory.labarchives.com/inventory/567051) | Millipore Sigma | C7698 |
| Benzonase Nuclease | Millipore Sigma | E1014-25KU |
| [Insulin(cattle)](https://inventory.labarchives.com/inventory/1257304) | MedChemExpress | HY-P1156 |
| Dexamethasone | Millipore Sigma | [D4902](https://www.sigmaaldrich.com/US/en/product/sigma/d4902) |
| [3-Isobutyl-1-methylxanthine](https://inventory.labarchives.com/inventory/1085254) | Millipore Sigma | I7018 |
| [Rosiglitazone](https://inventory.labarchives.com/inventory/1246963) | Millipore Sigma | R2408 |
| Tricine | Sigma | T0377 |
| [Noradrenaline tartrate](https://inventory.labarchives.com/inventory/1014046) | Millipore Sigma | N1100000 |
| [CL 316,243 hydrate](https://inventory.labarchives.com/inventory/1009118) | Millipore Sigma | C5976 |
| [Sucrose, Ultrapure Bioreagent, J.T. Baker™](https://inventory.labarchives.com/inventory/567054) | Fisher Scientific | 02-004-331 |
| Sodium chloride | Sigma | S9625 |
| Potassium chloride | Millipore Sigma | P5405 |
| Magnesium chloride hexahydrate | Millipore Sigma | M9272 |
| Acetone, HPLC Grade, ≥ 99.5%, LabChem™ | Fisher Scientific | LC104254 |
| Ethyl Ether | Sigma-Aldrich | EX0185-4 |
| Sodium dodecyl sulfate solution | Millipore Sigma | 71736 |
| Urea | Sigma-Aldrich | U5378 |
| Pierce™ Trypsin Protease, MS Grade | Thermo Scientific | 90057 |
| DNase I (Lyophilized) | Promega | Z3585 |
| [SuperScript™ IV Reverse Transcriptase](https://inventory.labarchives.com/inventory/1009122) | Thermo Fisher Scientific | 18090010 |
| Free Fatty Acid Assay Kit | LSBio | LS-K170-100 |
| Glycerol Assay Kit | Millipore Sigma | MAK117 |
| Pierce BCA Protein Assay Kit | Thermo Fisher Scientific | 23224 |
| [PARIS™ Kit](https://inventory.labarchives.com/inventory/1009127) | Invitrogen™ | AM1921 |
| Power SYBR Green PCR master Mix | Applied Biosystems | 4368708 |
| TaqMan reverse transcription reagents | Applied Biosystems | N8080234 |
| RNeasy Plus Universal Mini Kit | Qiagen | 73404 |
| SuperSignal™ West Pico Chemiluminescent Substrate | Thermo Fisher Scientific | 34580 |
| SuperSignal™ West Femto Maximum Sensitivity Substrate | Thermo Fisher Scientific | 34096 |
| TruSeq Stranded Total RNA | Illumina | 20020596 |
| Poly(A) Tail-Length Assay Kit | Invitrogen | 764551KT |
| DMEM High Glucose Medium | Corning | 10-013-CV |
| Nitrocellulose Membrane | Bio-Rad | 1620168 |
| 4–15% Criterion™ TGX™ Precast Midi Protein Gel, 26 well, 15 µl | Bio-Rad | 5671085 |
| 4–15% Criterion™ TGX™ Precast Midi Protein Gel, 18 well, 30 µl | Bio-Rad | 5671084 |
| 4–15% Mini-PROTEAN® TGX™ Precast Protein Gels, 10-well, 50 µl | Bio-Rad | 4561084 |
| 5% Criterion™ TBE Polyacrylamide Gel, 12+2 well, 45 µl 3450047 | Bio-Rad | 3450047 |
| InstantBlue® Coomassie Protein Stain (ISB1L) | Abcam | ab119211 |
| Pierce™ Protein G Magnetic Beads | Thermo Fisher Scientific | 88848 |
| [RNasin® Plus Ribonuclease Inhibitor](https://inventory.labarchives.com/inventory/390035) | Promega | N2615 |
| Heavy-isotope labeled peptide-DGLQESLPDNVHQVISGK (aa 96–113) | 21st Century Biochemicals | N/A |
| Heavy-isotope labeled peptide-YVDGGVSDNVPVLDAK (aa 163–179) | 21st Century Biochemicals | N/A |
| Heavy-isotope labeled peptide-STNFFHVNITNLSLR (aa 188–213) | 21st Century Biochemicals | N/A |

- 1. **Please provide the details of the corresponding methods author for the manuscript:**

| Helen H Hobbs  [Helen.Hobbs@UTSouthwestern.edu](mailto:Helen.Hobbs@UTSouthwestern.edu). |
| --- |

**2.0 Please confirm for randomised controlled trials all versions of the clinical protocol are included in the submission. These will be published online as supplementary information.**

|  |
| --- |
